# Supplementary material for: Multiplex knockout of trichome-regulating MYB duplicates in hybrid poplar using a single gRNA
Source: Plant Physiol. 2022 Mar 17;189(2):516–26. doi: 10.1093/plphys/kiac128 (PMC9157173; doi:10.1093/plphys/kiac128)
Supplement: kiac128_Supplementary_Data [file kiac128_supplementary_data.zip › SupplementalMaterialFinal.pdf]

Supplemental Table S1: Primers used in this study

Name	Purpose		Primer sequence (5' to 3')
p201N-Cas9	PCR of p201N-Cas9 vector backbone	F:	GAGTCGTATTACAATTCATGACTC
		R:	ATTTCATCGAAGAGATTAATATCGAATAATC
		cycle:	98°Cx30", [98°Cx15", 60°Cx20", 72°Cx8.5']x33 cycles, 72°Cx10', 15°C hold
MtU6	PCR of MtU6 promoter	F:	gatattaatctcttcgatgaaatttATGCCTATCTTATATGATCAATGAGG
		R:	AAGCCTACTGGTTCGCTTGAAG
		cycle:	98°Cx30", [98°Cx15", 60°Cx20", 72°Cx35"]x33 cycles, 72°Cx3', 15°C hold
Scaffold	PCR of synthetic scaffold	F:	GTTTTAGAGCTAGAAATAGCAAGTT
		R:	gtcatgaattgtaatacgactcAAAAAAAGCACCGACTCGGTG
		cycle:	98°Cx30", [98°Cx15", 60°Cx20", 72°Cx35"]x33 cycles, 72°Cx3', 15°C hold
gRNA (ΔG version)	gRNA oligos	F:	tcaagcgaaccagtaggcttGGAAGAGCTGTAGACTCAGG
		R:	aacttgctatttctagctctaaaaCCTGAGTCTACAGCTCTTCC
		cycle:	n/a (used directly for Gibson assembly)
gRNA (correct version)	gRNA oligos	F:	tcaagcgaaccagtaggcttGGAAGAGCTGTAGACTCAGG
		R:	aacttgctatttctagctctaaaacCCTGAGTCTACAGCTCTTCC
		cycle:	n/a (used directly for Gibson assembly)
MYB186/138/38	Amplicon sequencing	F:	cctacacgacgctcttccgatctCTTGATATCTGGCCGRAGGTAG
		R:	gttcagacgtgtgctcttccgatcGACKCCTGAAGAAGACCAGAAG
		cycle:	95°Cx3', [95°Cx30", 63°Cx45", 72°Cx25"]x25 cycles, 72°Cx3', 15°C hold
UBC, Potri.006G205700	Loading control for genomic PCR	F:	CTGAAGAAGGAGATGACARCMCCA
		R:	GCATCCCTTCAACACAGTTTCAMG
		cycle:	95°Cx3', [95°Cx30", 62°Cx30", 72°Cx45"]x30 cycles, 72°Cx3', 15°C hold
MYB186m1	Allele-specific PCR of MYB186.m1	F:	TACCCTCGGTTGGTCTGAG
		R:	AGTGGAAACCATGAGAGTGTG
		cycle:	95°Cx3', [95°Cx30", 62°Cx30", 72°Cx45"]x30 cycles, 72°Cx3', 15°C hold
MYB138m1	Allele-specific PCR of MYB138.m1	F:	TTGGTGCGTGCGTGTATAG
		R:	AGTGGAAACCATGAGAGTGTG
		cycle:	95°Cx3', [95°Cx30", 62°Cx30", 72°Cx45"]x30 cycles, 72°Cx3', 15°C hold
MYB186m2/ MYB138m1	Allele-specific PCR of MYB186.m2 and MYB138.m1	F:	GTTTGTCTCTCTCTCTCTCTCTCC
		R:	AGTGGAAACCATGAGAGTGTG
		cycle:	95°Cx3', [95°Cx30", 62°Cx30", 72°Cx45"]x30 cycles, 72°Cx3', 15°C hold
MYB138m2	Allele-specific PCR of MYB138.m2	F:	CGTGCGTGTGTGTGTGTATAG
		R:	AGTGGAAACCATGAGAGTGTG
		cycle:	95°Cx3', [95°Cx30", 62°Cx30", 72°Cx45"]x30 cycles, 72°Cx3', 15°C hold
MYB186a	Allele-specific PCR of MYB186.a	F:	TACCCTCGGTTGGTCTGAAATA
		R:	AGTGGAAACCATGAGATGAGAC
		cycle:	95°Cx3', [95°Cx30", 62°Cx30", 72°Cx45"]x30 cycles, 72°Cx3', 15°C hold
MYB138a	Allele-specific PCR of MYB138.a	F:	CCACGTTATAGTTTGTCTCTCTCTT
		R:	TATATCACCGCGAGGAACTACC
		cycle:	95°Cx3', [95°Cx30", 62°Cx30", 72°Cx45"]x30 cycles, 72°Cx3', 15°C hold
MYB186/MYB138	PCR of all 6 MYB186 and MYB138 alleles	F:	CCCCTGATATTTCTCCTTACYTTCC
		R:	TAGCAATGGCTGACCACCTG
		cycle:	95°Cx3', [95°Cx30", 62°Cx45", 72°Cx2']x30 cycles, 72°Cx3', 15°C hold

Lower case denotes homology sequence for Gibson assembly

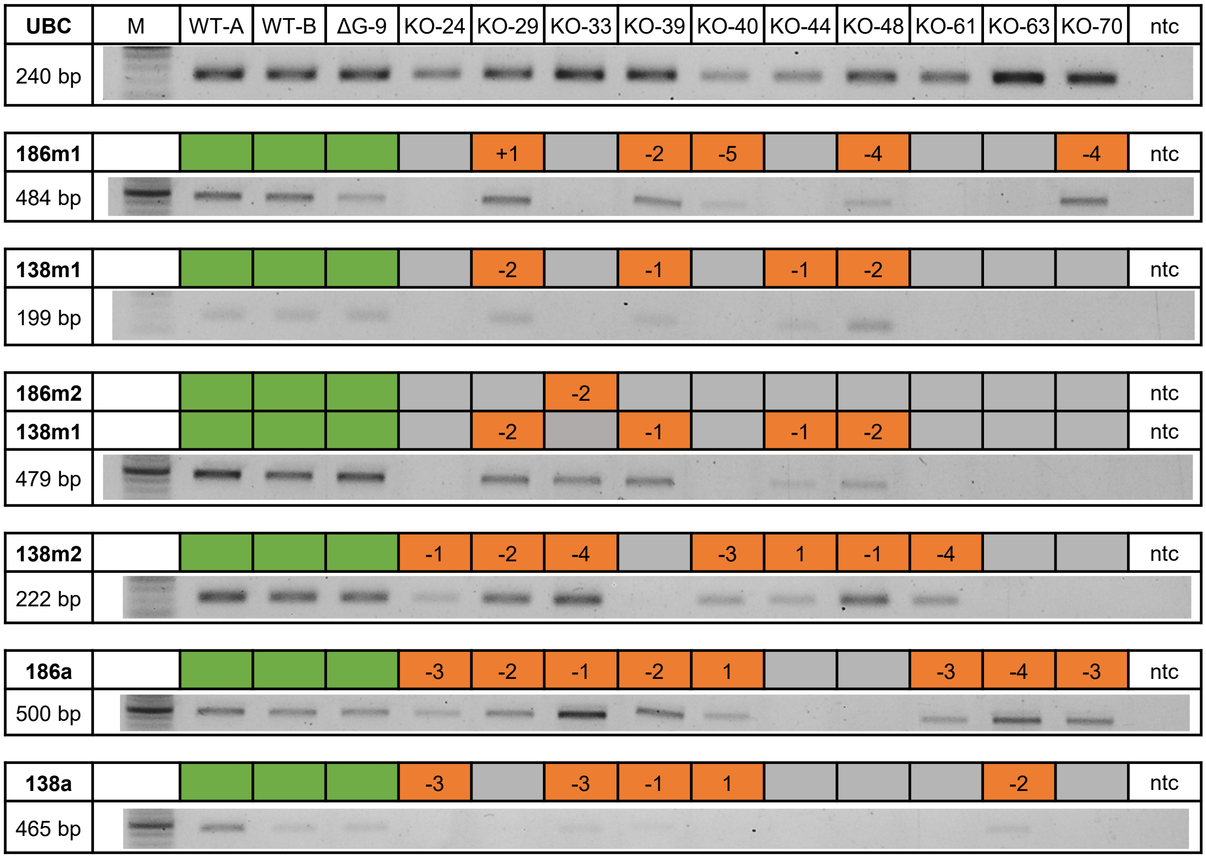

Supplemental Figure S1. PCR confirmation of NA alleles using allele-specific primers. Agarose gel images are aligned with mutation patterns determined by amplicon sequencing shown in Figure 3B. Allele-specific primers were not available for *MYB186m2*, and consensus primers for both *MYB186m2* and *MYB138m1* were used instead. Green, orange and grey colors indicate no editing, indel, and no amplification, respectively, for the given allele. PCR with ubiquitin-conjugating enzyme (UBC) primers was included as loading control. ntc, no template controls; M, molecular weight marker (NEB 100 bp DNA ladder).

Uncropped gel images for

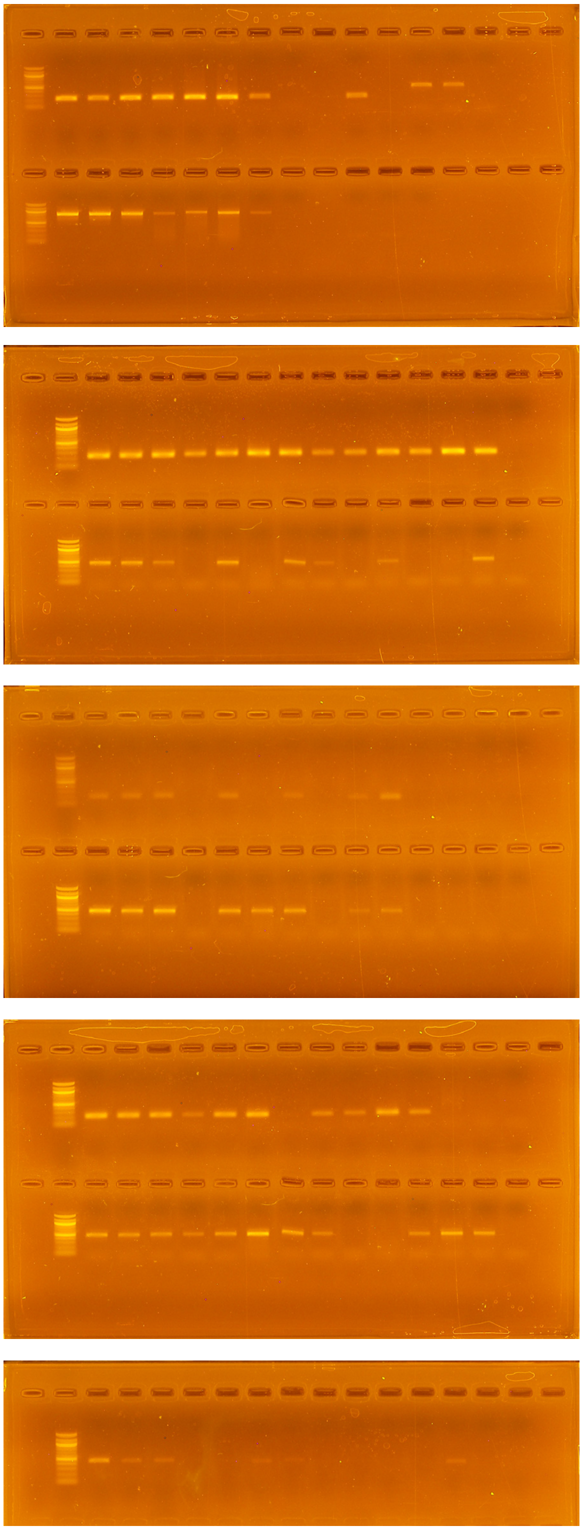

Fig. 4A (*UBC*)

Fig. 4A (*MYB*)

Fig. S1 (*UBC*)

Fig. S1 (*186m1*)

Fig. S1 (*138m1*)

Fig. S1 (*186m2/138m1*)

Fig. S1 (*138m2*)

Fig. S1 (*186a*)

Fig. S1 (*138a*)

Supplemental Figure S2. Original gel images.

```
186m2  GTCTCTCTCTCTCTCTCTCCGTTGGTCTGAGATATGGTTAAGTCTCAATGCTTTGAGAAGGTGGGACTGAAGAAAGGGCCATGGACTCCT
186m1  TGGCTATCATAGTACCCTCGGTTGGTCTGAGATATGGTCAAGTCTCAATGCTTTGAGAAGGTGGGACTGAAGAAAGGGCCATGGACTCCT
KO-5   TGGCTATCATAGTACCCTCGGTTGGTCTGAGATATGGTCAAGTCTCAATGCTTTGAGAAGGTGGGACTGAAGAAAGGGCCATGGACTCCT
186a   TGGCTATCATAGTACCCTCGGTTGGTCTGAAATATGGTAAAGTCTCAATGCTTTGAGAAGGTGGGACTGAAGAAAGGGCCATGGACTCCT
138m1  GTCTCTCTCTCTCTCTCTCCGTTGGTCTGAGATATGGTTAAGTCTCAATGCTTTGAGAAGGTGGGACTGAAGAAAGGGCCATGGACTCCT
138a   GTCTCTCTCTTTCTCTCTCCGTTGGTCTGAGATATGGTTAAGTCTCAATGCTTTGAGAAGGTGGGACTGAAGAAAGGGCCATGGACTCCT
138m2  ATCTCTCTCTTTCTCTCTCCGTTGGTCTGAGATATGGTCAAGTCTCAGTGCTTTGAGAAGGTGGGACTGAAGAAAGGGCCATGGACTCCT
KO-69  GTCTCTCTCTCTCTCTCTCCGTTGGTCTGAGATATGGTTAAGTCTCAATGCTTTGAGAAGGTGGGACTGAAGAAAGGGCCATGGACTCCT
                *      *** ********** ******* ******** ******************************************

186m2  GAAGAAGACCAGAAGCTTTTGGCTTACATCGAAGAGCATGGCCATGGAAGCTGGCGAGCCTTGCCTGCCAAAGCTGGTGATTTACATTAA
186m1  GAAGAAGACCAGAAGCTTTTGGCTTACATCGAAGAGCATGGCCATGGAAGCTGGCAAGCCTTGCCTGCCAAAGCTGGTGATTTACATTAA
KO-5   GAAGAAGACCAGAAGCTTTTGGCTTACATCGAAGAGCATGGCCATGGAAGCTGGCAAGCCTTGCCTGCCAAAGCTGGTGATTTACATTAA
186a   GAAGAAGACCAGAAGCTTTTGGCTTACGTCGAAGAGCATGGCCATGGAAGCTGGCAAGCCTTGCCTGCCAAAGCTGGTGATTTACATTAA
138m1  GAAGAAGACCAGAAGCTTTTGGCTTACATCGAAGAGCATGGCCATGGAAGCTGGCGAGCCTTGCCTGCCAAAGCTGGTGATTTACATTAA
138a   GAAGAAGACCAGAAGCTTTTGGCTTACATCGAAGAGCATGGCCATGGAAGCTGGCGAGCCTTGCCTGCCAAAGCTGGTGATTTACATTAA
138m2  GAAGAAGACCAGAAGCTTTTGGCTTACATCGAAGAGCATGGCCATGGAAGCTGGCGAGCCTTGCCTGCCAAAGCTGGTGATTTACATTAA
KO-69  GAAGAAGACCAGAAGCTTTTGGCTTACATCGAAGAGCATGGCCATGGAAGCTGGCGAGCCTTGCCTGCCAAAGCTGGTGATTTACATTAA
       *************************** *************************** **********************************

186m2  CAAACCCTTTATTTACTCCCAATTATTTATTCACTTGGTGCCCTAGTCTTTATTAAACGATAGCTTGATTGTTATCCCTTCCTTTTGTTT
186m1  CAAACCCTTTATTTACTCCCAATTATTTATTCACTTGGTGCCCTAGTCTTTATTAAACGATAGCTTGATTGTTATCCCTTCCTTTTGTTT
KO-5   CAAACCCTTTATTTACTCCCAATTATTTATTCACTTGGTGCCCTAGTCTTTATTAAACGATAGCTTGATTGTTATCCCTTCCTTTTGTTT
186a   CAAACCCTTTATTTACTCCCAATTATTTATTCACTTGGTGCCCTAGTCTTTATTAAACGATAGCTTGATTGTTATCCCTTCCTTTTGTTT
138m1  CAAACCCTTTATTTACTCCCAATTATTTATTCACTTGGTGCCCTAGTCTTTATTAAACGATAGCTTGATTGTTATCCCTTCCTTTTGTTT
138a   CAAACCCTTTATTTACTCCCAATTATTTATTCACCTGGTGCCCTAGTCTTTATTAAACGATAGCTTGATTGTTATCCCTTCCTTTTGTTT
138m2  CAAACCCTTTATTTACTCCCAATTATTTATTCACTTGGTGCCCTAGTCTTTATTAAACGATAGCTTGATTGTTATCCCTTCCTTTTGTTT
KO-69  CAAACCCTTTATTTACTCCCAATTATTTATTCACTTGGTGCCCTAGTCTTTATTAAACGATAGCTTGATTGTTATCCCTTCCTTTTGTTT
       ********************************** *******************************************************

186m2  TTTGGTGTGTGTGTGC--GTGTGTGTAGGACTTCAAAGATGCGGGAAGAGCTGTAGACTCAGGTGGACCAACTACCTTCGGCCAGATATC
186m1  TTTGGTGTGTGCGTGC--GTGTGTGTAGGACTTCAAAGATGCGGGAAGAGCTGTAGACTCAGGTGGACCAACTACCTTCGGCCAGATATC
KO-5   TTTGGTGTGTGCGTGC--GTGTGTGTAGGACTTCAAAGATGCGGGAAGAGCTGTAGA--CAGGTGGACCAACTACCTTCGGCCAGATATC
186a   TTTGGTGTGTGT------GTGTGTGTAGGACTTCAGAGATGCGGGAAGAGCTGTAGACTCAGGTGGACCAACTACCTTCGGCCAGATATC
138m1  TTTGGTGCGTGC--------GTGTATAGGACTTCAAAGATGCGGGAAGAGCTGTAGACTCAGGTGGACCAACTACCTTCGGCCAGATATC
138a   TTTGGTGTGTGT------GTGTGTGTAGGACTTCAGAGATGCGGGAAGAGCTGTAGACTCAGGTGGACCAACTACCTTCGGCCAGATATC
138m2  TTTGGTGCGTGCGTGTGTGTGTGTATAGGACTTCAAAGATGCGGGAAGAGCTGTAGACTCAGGTGGACCAACTACCTTCGGCCAGATATC
KO-69  TTTGGTGCGTGC--------GTGTATAGGACTTCAAAGATGCGGGAAGAGCTGTAGA--CAGGTGGACCAACTACCTTCGGCCAGATATC
       ******* ***         **** ********** *********************  *******************************

186m2  AAGAGAGGAAAGTTTAATTTGCAGGAAGAACAATCAATCATTCAGCTGCATGCTCTTCTTGGAAACAGGTG-------------------
186m1  AAGAGAGGAAAGTTTAATTTGCAGGAAGAACAATCAATCATTCAGCTGCATGCTCTTCTTGGAAACAGGTGAGATGGTAGTTCCTCGCGG
KO-5   AAGAGAGGAAAGTTTAATTTGCAGGAAGAACAATCAATCATTCAGCTGCATGCTCTTCTTGGAAACAGGTG-------------------
186a   AAGAGAGGAAAGTTTAATTTGCAGGAAGAACAATCAATCATTCAGCTGCATGCTCTTCTTGGAAACAGGTGAGATGGTAGTTCCTCGCGG
138m1  AAGAGAGGAAAGTTTAATTTGCAGGAAGAACAATCAATCATTCAGCTGCATGCTCTTCTTGGAAACAGGTG-------------------
138a   AAGAGAGGAAAGTTTAATTTGCAGGAAGAACAATCAATCATTCAGCTGCATGCTCTTCTTGGAAACAGGTGAGATGGTAGTTCCTTGCGG
138m2  AAGAGAGGAAAGTTTAATTTGCAGGAAGAACAATCAATCATTCAGCTGCATGCTCTTCTTGGAAACAGGTGAGATGGTAGTTCCTCGCGG
KO-69  AAGAGAGGAAAGTTTAATTTGCAGGAAGAACAATCAATCATTCAGCTGCATGCTCTTCTTGGAAACAGGTGAGATGGTAGTTCCTCGCGG
       ***********************************************************************

186m2  --ATATATAGGATCTTGAAATTGACGGTCACACT-------------------CTCATGGTTTCCACTTTGGTATTATTATTCCTTCGAT
186m1  TGATATATAGGATCTTGAAATTGACGGTCACACT-------------------CTCATGGTTTCCACTTTGGTATTATTATTCCTTCGAT
KO-5   --ATATATAGGATCTTGAAATTGACGGTCACACT-------------------CTCATGGTTTCCACTTTGGTATTATTATTCCTTCGAT
186a   TGATATATAGGATCTTGAAATTGACGGTCACACTCTCATATATGTGGTCTCATCTCATGGTTTCCACTTTGGTATTATTATTCCTTCGAT
138m1  --ATATATAGGATCTTGAAATTGACGGTCACACT-------------------CTCATGGTTTCCACTTTGGTATTATTATTCCTTCGAT
138a   TGATATATAGGATCTTGAAATTGACGGTCACACTCTCATATATGTGGTCTCATCTCATGGTTTCCACTTTGGTATTATTATTCCTTCGAT
138m2  TGATATATATGATCTTGAAATTGACGGTCACACT-------------------CTCATGGTTTCCACTTTGGTATTATTATTCCTTCGAT
KO-69  TGATATATATGATCTTGAAATTGACGGTCACACT-------------------CTCATGGTTTCCACTTTGGTATTATTATTCCTTCGAT
         ******* ************************                   *************************************

186m2  GATCTTTGGTTTTCGGATTTTGTGTCCACTCTCAGATCAGGAAAGGCACATGATGGTGTGAATGCGTTGAAAAGCATACTCTTAAAATTT
186m1  GATCTTTGGTTTTCGGATTTTGTGTCCGCTCTGAGATCAGGAAAGGCACATGATGGTGTGAATGCGTTGAAAATCATATTCTTAAAATTT
KO-5   GATCTTTGGTTTTCGGATTTTGTGTCCGCTCTCAGATCA---------------------------------------------------
186a   GATCTTTGGTTTCCGGATTTTGTGTCCGCTCTCAGATCAGGAAAGGCACATGATGGTGTGAATGCGTTGAAAATCATACTCTTAAAATTG
138m1  GATCTTTGGTTTTCGGATTTTGTGTCCGCTCTCAGATCAGGAAAGGCACATGATGGTGTGAATGCGTTGAAAATCATACTCTTAAAATTT
138a   GATCTTTGGTTTCCGGATTTTGTGTCCGCTCTCAGATCAGGAAAGGCACATGATGGTGTGAATGCGTTGAAAATCATACTCTTAAAATTT
138m2  TATCTTTGGTTTTCGGATTTTGTGTCCGCTCTCAGATCGG-AAAGGCACATGATGGTGTGAATGCGTTGAAAATCATACTCTTCAAATTT
KO-69  TATCTTTGGTTTTCGGATTTTGTGTCCGCTCTCAGATCGG-AAAGGCACATGATGGTGTGAATGCGTTGAAAATCATACTCTTC------
        *********** ************** **** *****
```

Supplemental Figure S3. Sequence alignment of wild type and fusion *MYB* alleles from KO-5 and KO-69.
KO-5 identifying SNPs (186m1 upstream of gRNA and 138m1 downstream of gRNA) are highlighted in blue. KO-69 identifying SNPs (138m1 upstream of gRNA and 138m2 downstream of gRNA) are highlighted in yellow. gRNA target site is shown in red with PAM sequence underlined.
